# A Need for Strategies to Reduce Alcohol Use After Metabolic and Bariatric Surgery: Technology-Based Intervention and Study Protocol for a Pilot Randomized Controlled Trial

**DOI:** 10.2196/80068

**Published:** 2026-01-07

**Authors:** Lisa R Miller-Matero, Celeste Pappas, Brittany Christopher, Roman Grossi, Alyssa Vanderziel, Nancy P Barnett, Roland S Moore, Sarah Bendit, Aaron Hamann, Arthur M Carlin, Oliver A Varban, Jordan M Braciszewski

**Affiliations:** 1 Behavioral Health Henry Ford Health System Detroit, MI United States; 2 Center for Health Policy and Health Services Research Henry Ford Health System Detroit, MI United States; 3 College of Human Medicine Michigan State University East Lansing, MI United States; 4 Department of Behavioral and Social Sciences, Center for Alcohol and Addiction Studies Brown University Providence, RI United States; 5 Pacific Institute For Research and Evaluation Berkeley, CA United States; 6 Department of Surgery Henry Ford Health System Detroit, MI United States

**Keywords:** metabolic and bariatric surgery, alcohol use, intervention, technology, motivational interviewing

## Abstract

**Background:**

Individuals who undergo metabolic and bariatric surgery (MBS) are at an increased risk of developing a postoperative alcohol use disorder. Therefore, preventive strategies are needed to mitigate this risk. A technology-based intervention has reduced alcohol use among other populations and could be used after MBS.

**Objective:**

This study aims to describe a technology-based intervention to reduce alcohol use after MBS and to report the study protocol.

**Methods:**

The intervention consists of 2 computerized brief intervention sessions followed by 3 months of daily, tailored, personalized SMS text messages in which the intervention content adapts to an individual’s readiness to change. The intervention will be tested in a pilot randomized controlled trial with assessments completed at baseline, 1 month after baseline, after the intervention, and at a 6-month follow-up.

**Results:**

This project was funded by the National Institute on Alcohol Abuse and Alcoholism in September 2020. Enrollment of 60 participants in the trial occurred from November 2023 to August 2024. We anticipate that this intervention will be feasible and acceptable. We hypothesize that the intervention group will have a lower proportion of individuals who report alcohol use at the postintervention and follow-up assessments. We also expect that those in the intervention group will show an increased rating for the importance of avoiding alcohol use. We plan to explore the direction of effects for other outcomes, including hazardous alcohol use, attitudes toward drinking, psychiatric symptoms, and eating and lifestyle behaviors.

**Conclusions:**

A technology-based approach could be a feasible and acceptable method of delivering preventive strategies to individuals after MBS. The long-term goal is to develop an effective, scalable, and sustainable intervention to mitigate the risk of hazardous alcohol use and alcohol use disorder after MBS.

**Trial Registration:**

ClinicalTrials.gov NCT04788316; https://clinicaltrials.gov/study/NCT04788316

**International Registered Report Identifier (IRRID):**

DERR1-10.2196/80068

## Introduction

Despite the efficacy of metabolic and bariatric surgery (MBS) for long-term weight loss and improvement in medical comorbidities [1-3], patients are at risk of postoperative alcohol use disorder (AUD) [4]. Although the preoperative prevalence of AUD is similar to that in the general population (ie, 6%-8%), the number of individuals with an AUD increases after MBS, and as many as 1 in 5 individuals have an AUD 5 years following MBS [4,5]. During the preoperative evaluation, patients seeking MBS are routinely assessed for a history of alcohol use and AUD and are provided with education about their increased risk of postoperative alcohol misuse and AUD [6]. Consequently, patients are counseled to remain abstinent or minimize postoperative alcohol use. Although patients recall receiving this education, most patients engage in postoperative alcohol use, which places them at risk for an AUD [7]. Thus, there is a need for more novel strategies to mitigate the risk of alcohol misuse and AUD after MBS.

One promising option for reducing the risk of AUD following MBS is delivering intervention content using technology. A systematic review found that various approaches were used to deliver education and intervention content after MBS, including online, web-based, and smartphone delivery [8]. Randomized controlled trials cited in this review suggested that these approaches have the potential to improve outcomes, including weight loss, disordered eating, and physical activity, although the sample sizes were small [8]. However, technology-based tools have not yet been used to reduce alcohol use after MBS. A technology-based intervention could be a reasonable approach to decrease postoperative alcohol use, given the success of technology-based approaches for other outcomes after MBS.

Although there are existing technology-based interventions to reduce alcohol use [9], these are not appropriate for use after MBS in their current form. First, existing interventions were developed and tested for individuals who were engaging in heavy alcohol use [9]. Given the high risk of developing a postoperative AUD, a prevention approach may be best so that we intervene with individuals before escalation to heavy alcohol use. Consequently, strategies to prevent reinitiation of postoperative drinking, or to decrease alcohol use soon after reinitiation, are needed. Intervention content that targets heavy drinking would not be appropriate for those who are not yet drinking or are drinking at lower levels. Second, patients who have undergone MBS may not be motivated to engage in an intensive intervention solely focused on alcohol use if they do not see themselves as having a problem [10]. A technology-based approach may be an appropriate option for delivery of strategies to reduce alcohol use after MBS, as it can offer a lower-intensity intervention, at a low cost, and be tailored to the MBS experience as well as personalized to the individual’s goals.

In this paper, we describe the study protocol of a pilot randomized controlled trial of a technology-based approach to reduce postoperative alcohol use, which includes computerized brief intervention (CBI) sessions and SMS text messaging. The goals of the pilot investigation are to study the feasibility, acceptability, and preliminary outcomes.

## Methods

### Description of the Intervention

The technology-based intervention is composed of 2 sessions of CBI, followed by daily SMS text messaging. Intervention content is grounded in motivational interviewing (MI), which is a patient-centered approach with the goal of eliciting behavior change [11,12]. MI is complementary to the transtheoretical model (TTM)—a theory positing that behavior change occurs in increments along stages—in that the health care provider assesses the patient’s stage of readiness and motivation to change, and intervenes accordingly [13]. MI is particularly effective at the initial stages of change within the TTM [14], which is relevant for many individuals who undergo MBS, as they may not be motivated to engage in an alcohol-related intervention if they have not developed a problem with alcohol.

In developing the intervention, we obtained feedback from 20 participants who underwent MBS and were engaging in regular postoperative alcohol use [15]. We then conducted an open trial with 10 participants in which they received the 2 CBI sessions and 1 month of SMS text messaging. On the basis of feedback from the interviews and open trial, we finalized the intervention content and delivery logistics.

### CBI Sessions

The CBI was developed using the Computerized Intervention Authoring System [16] and is delivered in a 2-session, web-based format, each lasting 10 to 15 minutes. A 3D, mobile, and emotionally expressive animated narrator reads all the presented material during the sessions. The narration has been found to be respectful, well-understood, and well-liked by other populations [17,18].

CBI session 1 begins with an importance ruler, in which participants are asked, “How important is it for you to avoid alcohol use?” and they respond on a scale of 0 (not at all important) to 10 (very important). The narrator then presents a brief educational component (facts about alcohol use), which incorporates a gender- and race-matched video vignette of a patient who develops an AUD after MBS. Education includes information tailored to this patient population regarding: (1) the new, increased risk for AUD after MBS, (2) reasons for the increased risk (ie, the physiological changes postsurgery and how alcohol is metabolized differently), (3) the health risks of alcohol use (eg, impact on nutrition, weight loss, and physical health), and (4) the “pros” of avoiding alcohol use (eg, impact of alcohol use on MBS, weight loss, mental health, success at work and school, and preventing injury). Participants are again asked about the importance of avoiding alcohol use using the importance ruler. Content is thereafter tailored to each individual’s importance rating. Participants who do not think it is important to avoid alcohol (indicated by scores 0-3) receive intervention content that mirrors phase I of MI (ie, building motivation to abstain or reduce drinking behavior) [12]. Those who are “not sure” (as measured by scores 4-7) receive content on identifying ways to know when it is time to change. For both groups (ie, 0-3 and 4-7), the narrator presents information to evoke change talk (ie, ideas that relate to making behavior changes) [11,12]. Specifically, the narrator asks questions, such as, “If you did to decide to make a change, what would be the best part about having cut down or quit?” “Picture things a year from now. How would you like them to turn out?” and “If you did decide to make a change, what are some reasons you might succeed?” For each of these prompts, participants are presented with myriad response options to choose from, including an open text box, and their responses are reflected by the narrator. A similar approach was successful in increasing confidence for change within a technology-based intervention [19]. Participants who endorse a high level of importance (scores 8-10) are asked about reasons for change, how they might change, and who might help them. All responses that reflect change talk (ie, interest in changing, identification of how they will know it is time to change, and reasons for change) are reinforced with affirmations and reflections. This combination of closed and open questions to elicit change talk, reflections of change talk, and affirmations is among the strongest predictors of further change and commitment talk within MI frameworks [20-23]. The selected change talk from this first CBI session provided by the participant is used in the subsequent SMS text messaging component, which takes place after CBI session 2.

Participants will receive a prompt to complete CBI session 2, 4 days after completing session 1. CBI session 2 begins with an introduction acknowledging that not everyone wants to change or believes that change is necessary. The narrator also states that we want to ensure that participants have the skills they may need in a difficult situation, should they want to make a change. A video vignette using the same gender- and race-matched character from CBI session 1 is presented, illustrating several different coping skills to achieve both weight loss and reductions in alcohol use. The participant then selects tailored coping strategies that they may find useful or want to learn more about. For example, patients can select to learn coping skills that are helpful for both overeating and drinking (ie, relaxation and managing emotional eating and cravings) [24]. The narrator then reviews difficult and risky situations that may arise (eg, “Everyone has the potential to run into difficult or risky situations. Sometimes, they may involve people in your life, and other times could result from feelings you have. Even if you don’t think you will soon experience a risky situation, which of these are you most likely to come across?”). The participant can choose from a list of difficult situations they feel are most appropriate for themselves (ie, social pressure, managing difficult emotions, etc). The narrator reflects on the choices selected and acknowledges that it can be helpful to be prepared even if a difficult event is not anticipated. The narrator offers ideas on how to handle the difficult situations and guides the participant through the development of a change plan. After finishing both CBI sessions, participants are asked to provide 1 final importance ruler score, which is used to tailor the SMS text messaging component of the intervention.

### SMS Text Messaging

On the basis of feedback from participants in the open trial, daily text messages will be delivered for 3 months. Using results from the CBI, text messaging is personalized. Messages are concise and focused, as both brevity (<160 characters) and immediacy are essential for maximal impact [25]. The content of most of the alcohol-related text messages was adapted from messages used in previous studies [26,27], as well as being theoretically grounded in MI [12] and the TTM [10]. Additional messages were developed that are specific to the MBS experience. Examples of text messages are presented in Textbox 1.

**Examples of SMS text messages. Participants will receive messages tailored to their rating of importance to avoid alcohol on a 0 to 10 scale as follows: low importance (0-3 rating), moderate importance (4-7 rating), and high importance (8-10 rating). All participants will receive personalized goal-related and affirmation text messages.**

**Text messages based on the rating of the importance of avoiding alcohol use**

- Low importance
  If you decided to be alcohol free, what would be the biggest obstacle?
  Staying away from alcohol is hard. What’s the hardest thing for you?
  If you decided not to drink, what’s the worst that can happen? What’s the best that could happen?
- Moderate importance
  Just considering change is a step in a healthy direction.
  What goals do you have for the next years? How does alcohol fit into those goals?
  Like food, alcohol affects chemical pathways in the brain that control reward and pleasure.
- High importance
  Remind yourself that you can choose to drink or not. Make a promise to yourself to NOT drink and stick with your decision.
  Being around other people who are drinking is hard! Think about how you are going to handle those situations before they occur.
  When you get the urge to drink alcohol, distract yourself with something else you enjoy.

**Examples of nonalcohol text messages**

- One of the most helpful things after surgery is to track your food and liquids. Did you track today?
- Eating slowly allows your brain to register you are full and you may enjoy your food more.
- Parking further away from a store or doing some exercises during a commercial break are great ways to add to your physical activity.
- We all eat things we did not intend to. Getting back on track is a success.

**Examples of personalized goal-related text messages**

- You mentioned that alcohol could affect your weight loss journey. That could be a powerful motivator!
- You don’t want to develop a problem with alcohol later on. That is a good reason to minimize your alcohol use.
- A strategy you find helpful is engaging in positive, fun activities, such as music, art, dancing, or exercise.

**Examples of personalized affirmation text messages**

- You have not used alcohol since undergoing bariatric surgery. This is something to be proud of!
- You only drank 1 day last week, which was fewer than the 3 days you drank in the week before. Great job!
- You had two weeks in a row where you were alcohol free. It seems you have found helpful ways to avoid drinking!

SMS text messages are tailored to each participant’s level of importance to avoid alcohol. Initially, participants will receive messages that correspond with their level of importance rating made at the end of the CBI sessions. Participants will also respond to weekly “poll questions” to assess drinking frequency as well as the importance of avoiding alcohol use. On the basis of their responses, algorithms will determine the tailored messages each participant receives, allowing for fluid change in messages received throughout the 3-month duration of text messages. More specifically, messages will be delivered in 1 of the following 3 ways: those who, at the end of the CBI, indicate a low importance in avoiding alcohol use (ie, scores between 0-3; TTM: precontemplation) will receive message content that helps develop discrepancy between behavior, goals, and values [11,12]. Messages for participants with a moderate level of importance (ie, importance scores between 4-7; TTM: contemplation) will be more motivational in content. Those reporting a high level of importance (ie, scores between 8-10; TTM: preparation and action) will receive messages that include advice and tips. All participants will receive messages that are information-based, and they will also receive content specific to MBS that is nonalcohol focused, based on feedback received in our interviews and open trial.

In addition to the text messages based on their importance rating, participants will also receive personalized text messages twice per week. Selected change talk provided by participants during the CBI will be used to remind participants of the goals they set for themselves. For example, participants are asked to provide reasons for change, people who could help them change, change strategies, events that would precipitate change, and the best parts about potential change. We capture these entries verbatim and provide text messages using the participants’ language. Participants also receive personalized affirmation text messages (ie, positive feedback) when they reduce their drinking or report abstinence, which aligns with MI strategies [11,12].

### Study Protocol

#### Ethical Considerations

This study received funding from the National Institute on Alcohol Abuse and Alcoholism (grant R34 AA027775) and is registered on clinicaltrials.gov (NCT04788316). It is also approved by the health care system’s institutional review board (15583). All participants will provide informed consent before engaging in study activities. To protect privacy, participants needed to consent to have their data shared in a data repository. Participants who did not consent to this will not have their data shared. No identifying information of any participant will be shared or published.

#### Participants and Procedures

We plan to enroll 60 participants who underwent MBS 3 and 18 months before the invitation to participate in the study. This time frame was selected to engage patients who have not yet begun postoperative drinking (most reinitiate between 6 and 12 months postoperatively) [7] and to also engage individuals who began drinking, but are unlikely to have already developed AUD because the risk increases approximately 2 years postoperatively [4]. Patients who underwent MBS at a single institution within this time frame will be sent an email invitation to complete a web-based eligibility survey. Individuals will be eligible to participate if they engaged in alcohol use before MBS, have access to the internet to complete the CBI, and have a cellular phone that can receive SMS text messages. Eligible individuals will be contacted by our study team and provided with a detailed description of the study. Patients will be allowed to ask any questions to the study team and will provide a signature to document their informed consent. Once patients provide informed consent, participants will complete their baseline assessment through a web-based, secure, and Health Insurance Portability and Accountability Act–compliant program (Research Electronic Data Capture). Participants will then be randomized to the intervention group or the treatment-as-usual control group (ie, MBS-related follow-up care) using urn randomization, separating participants by sex and time elapsed since MBS. Participants will complete assessments 1 month after baseline, at the postintervention evaluation (ie, 3 months following baseline), and at the 6-month follow-up through Research Electronic Data Capture. Participants randomized to the intervention group will also complete an exit interview to obtain feedback about the intervention. Participants will be compensated US $50 for each assessment completed.

#### Measures

##### Demographics and Background Information

Information collected at baseline will include age, sex and gender, race, weight and BMI, education level, medications, history of psychological symptoms, history of psychological treatment, and history of substance use and substance use treatment.

##### Alcohol Use

We will measure past 30-day alcohol use, similar to other studies, using adapted items from the Behavioral Risk Factor Surveillance System Questionnaire [28]. Participants will be shown a diagram and description of a standard drink and report the number of days they consumed any alcohol in the past 30 days (0-30). The primary outcome is drinking status (ie, whether alcohol was consumed in the past 30 d). For those who endorse at least 1 drinking day, they will also report on the number of average standard drinks per day, the highest number of drinks in a day, the number of days they consumed 4 or more drinks, and the number of days they perceived that they were drunk. The presence of hazardous alcohol use will be measured with the Alcohol Use Disorder Identification Test-Consumption [29]. This version was chosen because it is shorter than the original Alcohol Use Disorder Identification Test, while still providing a reliable assessment of drinking behavior [29,30]. An Alcohol Use Disorder Identification Test-Consumption score of 3 or greater for women and 4 or greater for men indicates high risk for hazardous drinking.

##### Other Alcohol-Related Measures

Using the importance ruler, participants will provide their rating of the importance of avoiding alcohol on a scale from 0 (not at all important) to 10 (very important). The Drinking Motives Questionnaire-Revised [31] assesses respondents’ reasons for drinking across 4 categories: conformity, coping, enhancement, and social. Each subscale consists of 5 items with responses ranging from 1 (strongly disagree) to 4 (strongly agree).

##### Psychiatric Symptoms

We will measure depression with the Patient Health Questionnaire-8 depression scale, which is a widely used, validated measure [32]. We will also assess anxiety with the Generalized Anxiety Disorder-7, which is commonly used to assess symptoms of anxiety [33]. Scores greater than or equal to 10 indicate a high likelihood of current depression or anxiety, respectively.

##### Eating and Lifestyle Behaviors

To explore the potential impact of the intervention on other important MBS-related outcomes, we will include measures related to eating and lifestyle behaviors. We will track the symptoms of food addiction using the Yale Food Addiction Scale 2.0 [34]. We will measure other eating behaviors with the Emotional Eating Scale [35] and the Eating Disorder Examination-Questionnaire [36]. We will also query about the frequency of lifestyle behaviors, including the use of a food diary, exercise, mindful eating, self-weighing, and obtaining 7 to 9 hours of sleep.

##### Study Feasibility

Feasibility will be assessed in several ways. First, we will calculate eligibility rates as the number who meet eligibility criteria divided by the number screened. Second, enrollment rates will be defined as the number of participants who enroll divided by the number eligible. To improve future recruitment strategies, we will record the reasons for nonenrollment. Finally, retention will be calculated using the rate of follow-up completion, as well as a determination of whether there was differential completion between the control and intervention groups.

##### Intervention Adherence

We will examine engagement with the intervention by examining the initiation and completion of CBI sessions and the response rate to the weekly poll questions.

##### Intervention Acceptability

We will use the Usability and Satisfaction Assessment to capture quantitative data on usability, overall satisfaction, helpfulness, content relevance, and use of intervention skills [37]. Items will query these areas for both the CBI sessions and SMS text messaging to examine acceptability of these components separately (ie, “I liked the 2-session computerized brief intervention,” “Is the content in the text messages relevant?” and “How helpful do you think the text messages were?”). The items will be rated on a 5-point scale and could be considered positive (ie, acceptable), neutral, or negative (ie, unacceptable). Exit interviews will provide qualitative feedback on intervention acceptability.

#### Analytic Plan

We will examine the frequencies of our feasibility, adherence, and acceptability variables. Although the primary focus is on feasibility and acceptability, we also plan to examine the direction of effects on outcomes. Our primary outcome is alcohol use. We will conduct chi-square and logistic regressions for categorical outcomes, and analysis of covariance for continuous variables (controlling for baseline measurements) to examine differences in outcomes between the intervention and control groups at the postintervention evaluation. For the 6-month follow-up, logistic regression will be used for the categorical outcome (eg, any alcohol use at 30 days), and hierarchical linear modeling will be used to examine repeated measurements of alcohol use quantity and frequency, covarying for baseline levels of those variables and demographics. Hierarchical linear modeling is optimal for analyzing longitudinal data, as it allows for control over missing data and comparison of more than 2 waves of data [38,39].

## Results

This project was funded by the National Institute on Alcohol Abuse and Alcoholism on September 20, 2020. After completing patient interviews and an open trial to refine the intervention, recruitment for the pilot randomized controlled trial began on November 8, 2023, and the final participant was enrolled on August 14, 2024. A total of 60 patients were enrolled.

Because this is a pilot trial, the primary focus will be on the feasibility of the study protocol, adherence to the intervention, and acceptability of intervention content. We anticipate having an enrollment rate of 50% and at least 75% who complete each assessment. We anticipate that at least 75% of the participants will initiate the intervention (ie, complete at least 1 CBI session). To measure adherence and engagement in the intervention content, we anticipate that at least 75% of the weekly SMS text messages will be responded to and that at least 75% of the participants will complete the intervention (ie, complete both CBI sessions and continue to receive the 3 months of SMS text messaging). We also expect that the majority (>50%) of participants will rate each of the acceptability items as positive and that at least 75% of the participants will believe that other patients would use the intervention after MBS. We have set these benchmarks, as they are deemed reasonable definitions of success for pilot trials [40].

We hypothesize that those in the intervention group will have a lower proportion of individuals engaging in alcohol use at the postintervention and follow-up assessments compared to the control group. We also expect that individuals in the intervention group will report greater importance to avoid alcohol use compared to the control group at the postintervention evaluation and follow-up. Because this is a pilot study and we may not be powered to detect between-group differences, we will also explore changes within groups to determine whether there is potential for an effect (ie, a significant increase in their rating on the importance of avoiding alcohol use among the intervention group). We will explore potential differences between the intervention and control groups for our other outcomes, including proportion of individuals with hazardous drinking, attitudes toward drinking, psychiatric symptoms, and eating and lifestyle behaviors. Although we do not expect statistically significant differences given the small sample size, we hypothesize that those in the intervention group will show a direction toward improvement, and we will examine the effect sizes of the intervention.

## Discussion

### Anticipated Findings

The goal of this pilot randomized controlled trial is to examine the feasibility and acceptability of a multicomponent technology-based intervention and to examine the direction of intervention effects. We anticipate that this study protocol will be feasible, that participants will engage with and adhere to the components in the technology-based intervention, and that participants will find the intervention content acceptable. We also anticipate that those randomized to the intervention group will have lower alcohol use than those in the control group. Other technology-based interventions for reducing substance use have been feasible, acceptable, and successful at reducing substance use, so this approach will likely translate to individuals who undergo MBS [41]. However, most interventions for substance use are targeted at individuals who have developed substance abuse; therefore, this approach is unique in that the goal is preventive. Moreover, given that the risk of AUD after MBS increases between 1 and 2 years postoperatively, delivering content to the population between 3 and 18 months postoperatively could capture the most vulnerable period for reinitiation and escalation of alcohol use [4].

### Strengths and Limitations

This study has several strengths, including the preventive approach to mitigating AUD after MBS; the use of a control group as a comparison to treatment-as-usual; and the collection of data that includes feasibility, acceptability, and preliminary outcomes. However, it is important to note several limitations. First, this is a pilot study with a small sample size. Consequently, the study may not be appropriately powered to detect changes in alcohol use. Therefore, the goal is to examine the direction of the effects to determine whether a future, fully powered trial is worthwhile. Second, the length of follow-up is short; therefore, the ability to prevent the development of an AUD cannot be assessed. Finally, alcohol use is measured by self-report, and it is possible that patients may minimize their use or report their use inaccurately.

### Conclusions and Future Directions

Patients who undergo MBS are at an increased risk of developing a postoperative AUD. Previous research has suggested a need for strategies to reduce postoperative alcohol use to mitigate the risk of an AUD. A technology-based approach could be a feasible and acceptable method of delivering preventive strategies to individuals after MBS. If successful, this project will provide the necessary support to conduct a fully powered randomized controlled trial to examine its efficacy in reducing alcohol use and with longer-term follow-up to determine whether it can reduce the incidence of AUD. Consequently, the next step after this pilot study is to conduct a randomized trial of this technology-based intervention compared to a control group. We plan to determine whether this intervention could prevent the reinitiation of postoperative alcohol use and reduce alcohol use among those who did reinitiate drinking after MBS. We also plan to test mediators and moderators in future work, as well as to examine the factors necessary for the successful implementation with MBS programs. Our long-term goal in this line of research is to develop an effective program that is scalable and sustainable within MBS programs to mitigate the risk of hazardous alcohol use and AUD.

## Data Availability

Data sharing is not applicable to this paper as no datasets were generated or analyzed during this study.

## Appendix

Multimedia Appendix 1 Peer review report by AA-3 Clinical, Treatment and Health Services Research Review Subcommittee, National Institute on Alcohol Abuse and Alcoholism Initial Review Group (National Institutes of Health, USA).

## Abbreviations

AUD: alcohol use disorder
CBI: computerized brief intervention
MBS: metabolic and bariatric surgery
MI: motivational interviewing
TTM: transtheoretical model
