# Supplementary material for: A Need for Strategies to Reduce Alcohol Use After Metabolic and Bariatric Surgery: Technology-Based Intervention and Study Protocol for a Pilot Randomized Controlled Trial
Source: JMIR Res Protoc. 2026 Jan 7;15:e80068. doi: 10.2196/80068 (PMC12824574; doi:10.2196/80068)
Supplement: Multimedia Appendix 1 [file resprot_v15i1e80068_app1.pdf]

SUMMARY STATEMENT
(Privileged Communication)

PROGRAM CONTACT:
Anita Bechtholt
301-480-8368
anita.bechtholt@nih.gov

Release Date: **03/25/2020**
Revised Date:

Application Number: **1 R34 AA027775-01A1**

Principal Investigators (Listed Alphabetically):

BRACISZEWSKI, JORDAN MICHEL
MATERO, LISA (Contact)

Applicant Organization: Henry Ford Health System

Review Group: **AA-3**
Clinical, Treatment and Health Services Research Review Subcommittee

Meeting Date: **03/12/2020**
Council: **MAY 2020**
Requested Start: **07/01/2020**
RFA/PA: **PA18-775**
PCC: **AC A**

Project Title: **A Technology-based Intervention to Reduce Alcohol Use after Bariatric Surgery**

SRG Action: **Impact Score:33**
Next Steps: **Visit https://grants.nih.gov/grants/next_steps.htm**
Human Subjects: 48-At time of award, restrictions will apply
Animal Subjects: 10-No live vertebrate animals involved for competing appl.
Gender: 1A-Both genders, scientifically acceptable
Minority: 1A-Minorities and non-minorities, scientifically acceptable
Age: 3A-No children included, scientifically acceptable

Project Year	Direct Costs Requested	Estimated Total Cost
1	150,000	225,750
2	150,000	225,750
3	150,000	225,750
TOTAL	450,000	677,250

ADMINISTRATIVE BUDGET NOTE: The budget shown is the requested budget and has not been adjusted to reflect any recommendations made by reviewers. If an award is planned, the costs will be calculated by Institute grants management staff based on the recommendations outlined below in the COMMITTEE BUDGET RECOMMENDATIONS section.

1R34AA027775-01A1 Matero, Lisa

PROTECTION OF HUMAN SUBJECTS UNACCEPTABLE

RESUME AND SUMMARY OF DISCUSSION: This amended R34 will evaluate the efficiency of an alcohol prevention approach on patients who had undergone bariatric surgery. The outcome of these studies has a significant potential to improve human health since the knowledge gained could have impact on reducing alcohol use in this population. The applicants are experts in their respective fields and have a strong track record of productivity. The collaborators assembled are highly experienced and their contribution to the proposed research program is important, and the scientific environment to conduct the proposed research is excellent. The MPI plan and overall study organization are clearly developed and takes advantage of the complementary expertise of the two study leaders. The applicant has been partially responsive to prior critiques; however, several major weaknesses still remain, which dampened enthusiasm. The experimental design does not involve innovative approaches or conceptual innovation. Most of the basic brief intervention components are basically standard and not cutting-edge interventions. It is not clear the rationale on recruiting individuals after six months of the surgery, as these patients have already initiated a drinking behavior change. The application also does not describe the current eating behavior of this population. The lack of clear information about these two parameters can affect the analysis and interpretation of results. Following the discussion, the panel rated the proposal in the very good to excellent range.

DESCRIPTION: Despite bariatric surgery being the most effective weight loss intervention for patients who are severely obese, as many as 1 in 5 patients will develop an alcohol use disorder after their surgery. Changes in metabolism, hormone levels, and behavior as a result of bariatric surgery alter the rewarding effects of alcohol while concurrently changing its absorption rate, putting patients at significantly elevated risk of hazardous drinking. Simply providing education to this vulnerable patient population about post-surgical risks has not been sufficient to reduce alcohol use, yet comprehensive in-person interventions are met with significant challenges, including hours-long distances between patients and their bariatric surgery programs. Thus, our long-term goal is to increase access to an empirically supported intervention for reducing alcohol use among patients who undergo bariatric surgery by leveraging technology. Our intervention, rooted in motivational interviewing and the transtheoretical model, is a two-session computerized brief intervention (CBI), supplemented by six months of tailored text messaging based on participants' CBI results and subsequent fluctuations in their readiness to change. The purpose of the proposed study is to optimize this technology-based intervention for patients who undergo bariatric surgery and to examine feasibility and acceptability of the intervention. In the first phase, patient interviews (n= 20) will be utilized to identify preferences for intervention content and treatment delivery. Ten patients will then participate in an open trial of the intervention, which will be subsequently revised based on feedback from these patients. In Phase 2, patients (N = 60) will be recruited between 3 and 6 months following bariatric surgery and randomized to the intervention or treatment as usual control group. All patients will complete baseline questionnaires and at 1, 3, 6, and 9-month post-assessments. We expect that this intervention will be both feasible and acceptable to patients. Results will be used as preliminary data to inform a large, fully powered clinical trial to test the larger efficacy of this intervention. Although primary outcomes focus on feasibility and acceptability, we also expect that patients assigned to the intervention will have a longer time to their first post-surgical drink, report more days of abstinence, fewer drinks per drinking day, and a lower prevalence of alcohol use disorder after bariatric surgery compared to controls. This project is innovative because it expands upon existing interventions for bariatric surgery patients by implementing evidence-based strategies for alcohol use. By utilizing a technology-based approach, we can also reach a larger number of patients to prevent initiation of drinking, reduce current alcohol use, and facilitate better engagement in care, should individuals opt into traditional treatment approaches. The proposed line of research is significant and relevant to NIH's mission because the intervention is expected to reduce the likelihood that patients will develop an alcohol use disorder following bariatric

surgery. Given the potential of wide dissemination at low cost, the proposed study has high potential public health and clinical significance.

PUBLIC HEALTH RELEVANCE: Although bariatric surgery is an effective method for substantial weight loss, more than 20% of individuals experience an alcohol use disorder after surgery due to metabolic, hormone, and behavioral changes. Despite significant risks of problematic alcohol use and a growing population of individuals undergoing bariatric surgery, current clinical practice involves only education about the hazards of post-surgical alcohol consumption. The proposed study moves the field forward and impacts public health by leveraging technology to deliver a theoretically based, empirically-supported intervention to delay the initiation of alcohol use, reduce the amount of alcohol consumed, and prevent the development of an alcohol use disorder among bariatric surgery patients.

CRITIQUE 1

Significance: 3
Investigator(s): 2
Innovation: 3
Approach: 4
Environment: 1

Overall Impact: This revised application sought to develop, and collect pilot and feasibility data on, a technology-based intervention targeting the prevention and reduction of alcohol use among individuals who have recently undergone bariatric surgery. The significance of this proposal lies in the increased risk for alcohol use disorder (AUD) and alcohol related problems experienced following surgery. The aims follow the guidelines for Stage 1 intervention development. A computerized brief intervention (CBI), supplemented by six months of daily personalized text messaging based on participants' motivation for change and subsequent fluctuations in their desire to change will be compared to treatment as usual (pre-surgery psychoeducation). The revised application was responsive to many of the critiques from the previous review. The research team has the required expertise and the environment is ideal to complete the proposed work, as well as take it to the next step if successful. Despite the many improvements in the research plan, there was a concern that the focus on readiness/or stage of change seemed to be mismatched with the alcohol use status of participants. That is, they will have already initiated drinking cessation, and will have been abstinent since their surgery 3-6 months before the trial. The application did not consider whether/how the proposed interventions that hold promise for initiating drinking behavior change would target the patients' ability to sustain their drinking behavior change.

1. Significance

Strengths

- Bariatric surgery for weight loss is associated with an increased risk of problem drinking and developing an alcohol use disorder (AUD), likely due to both physiological and psychological mechanisms.
- A technology-based intervention aimed at delaying initiation of, or promoting reductions in, alcohol use was well-suited to the needs of post-bariatric surgery patients.

Weaknesses

- The focus on attitudes towards alcohol as mediators (intervention targets) of the relation between bariatric surgery and increased risk for alcohol use seemed to be inconsistent with the motivational (coping, enhancement, social reasons for use) conceptual model endorsed. Yet, it appeared that attitudes towards drinking were planned to be measured with a drinking

motives/reasons for drinking measure, so perhaps this was a semantic issue. Attitudes and motives are different constructs.

2. Investigator(s)

Strengths

- Drs. Lisa Matero and Jordan Braciszewski will serve as Multi-PIs. The leadership plan clearly differentiated their complimentary areas of expertise needed for the proposed studies.
- Dr. Matero has expertise in implementing research on post-surgery alcohol use and in examining predictors of AUD.
- Multi-PI Dr. Braciszewski has substantial background in technology-based alcohol interventions, and the team is further strengthened by the addition of Dr. Burnett as a consultant.
- Dr. Nancy Burnett was added as a consultant to compliment expertise in developing and implementing brief interventions in health care settings.

Weaknesses

- None noted.

3. Innovation

Strengths

- There are no current empirically support approaches to prevent or reduce alcohol use in the post-bariatric surgery population, a notable proportion of which experiences a surgery-linked increase in risk for AUD.
- The adaptive nature of the text messaging depending on readiness for change appeared to be novel.

Weaknesses

- Although technology-based interventions for alcohol use have not been tailored to the bariatric surgery population, many of the basic brief intervention components are not in themselves novel.

4. Approach

Strengths

- The application Aims follow the guidelines for Stage 1 intervention development guidelines.
- Phase 1 was Optimization of the intervention using patient interviews (N = 20) to evaluate and refine the intervention. An open trial with 10 patients will be used to assess feasibility and refine the protocol based on patient feedback. Phase 2 was a randomized controlled trial (N= 60) to pilot test acceptability, feasibility, and direction of changes on outcomes for the new intervention compared to a treatment-as-usual control group.
- Open trial and RCT participants will be non-drinkers at baseline with a history of pre-surgery drinking but never met AUD criteria.
- The CBI Session 1 included a combination of closed and open questions to elicit change talk, reflections of change talk, and affirmations, which will be incorporated to personalize the text messaging component. CBI Session 2 was coping skills focused to achieve weight loss and reductions in alcohol use and develop tailored coping strategies. Boosters will be provided by the content of daily test messaging (1 mon Open trial; 6 mon RCT) that be adaptive in that it will

be matched to the participants' scores on the 'Importance Ruler' that assesses their stage of change on a weekly basis.

Weaknesses

- There is a question about the focus on alcohol use behavior change per se, as participants will be former drinkers who are current non-drinkers at the study's baseline. Thus, at the start of the trials, they have already initiated drinking behavior change. This raised the question of whether the intervention should focus on supporting *maintenance of drinking behavior change* (i.e., sustaining non-drinking), rather than readiness for initiating behavior change.
- On the other hand, it was not clear where participants would be on the continuum of weight loss/eating behavior change, so the combination of this target with drinking behavior in CBI Session 2 may be complicated.
- The treatment- as-usual control was standard psychoeducation administered pre-surgery, which is known to be minimally active; was there consideration to comparing the target intervention to a but less resource intensive electronic intervention post-surgery that has already been validated in a non-surgical population?

5. Environment

Strengths

- The application will be developed and tested at the Henry Ford Health System, Detroit, Michigan. The Henry Ford Health System Bariatric Surgery Program is designated as a Center of Excellence and projected to complete 700 surgeries annually.
- The Henry Ford Health System is part of a large network of partner health care systems called the Mental Health Research Network (MHRN; U19MH092201), a consortium of 14 health systems and over 15 million patients, which will provide a valuable environment to a future multi-site study, if supported by the current study.

Weaknesses

- None noted.

Study Timeline

Strengths

- Appeared to be feasible.

Weaknesses

- None noted.

Protections for Human Subjects

Unacceptable Risks and/or Inadequate Protections

- Secure and HIPPA compliant electronic data collection methods.
- Appropriate data storage.
- Referrals will be made for participants who develop serious mental health concerns.

Data and Safety Monitoring Plan (Applicable for Clinical Trials Only):

Unacceptable

- No procedures noted for reporting unexpected or adverse events.

Inclusion Plans

- Sex/Gender: Distribution justified scientifically.
- Race/Ethnicity: Distribution justified scientifically.
- Inclusion/Exclusion Based on Age: Distribution justified scientifically.
- Although the patient population of bariatric surgery is 82% women, recruitment will be targeted to include 50% men. This appeared feasible given the small sample size to be recruited and the large number of bariatric surgeries performed yearly at the recruitment site.
- Recruitment will be targeted to recruit equal numbers of White and racial minority participants, primarily African American.
- Bariatric surgery is not offered by the service to persons under 18 years of age.

Resubmission

- The investigators sought to broadly respond to the concerns raised in the previous review.
- Changes in attitudes about alcohol use was put forth in the conceptual model as the mediator of intervention effects (although attitudes were operationalized as motives).
- In considering intervention development compared to existing efficacious interventions, the investigators pointed to the sudden change in AUD risk level that occurs following bariatric surgery, which is unique to this population.
- Benchmarks recommended by SAMHSA to identify successful feasibility and acceptability to support a future R01 were added.
- Increased opportunities for patients to engage in change talk.
- Shift from sole focus on abstinence outcome to risk reduction; outcome variables include time to first post-surgery drink, number of days abstinent, and drinks per drinking day.
- Clarified unique goals of the open trial (intervention content acceptability and procedures) vs the pilot RCT (retention, intervention delivery, and intervention outcomes).
- Clarification of follow-up timeline: last follow-up 3 months post text messaging component of intervention but near 9 mon post CBI component.
- Nancy Barnett added as expert consultant in developing and implementing brief interventions for alcohol use in health care settings.
- No consideration of suggestion to provide cell phones to those who would be excluded for not having one, although this was estimated to be a very small number.

Resource Sharing Plans:

Acceptable

Authentication of Key Biological and/or Chemical Resources:

Not Applicable (No Relevant Resources)

Budget and Period of Support

Recommend as Requested

CRITIQUE 2

Significance: 3
Investigator(s): 1
Innovation: 4
Approach: 2
Environment: 2

Overall Impact: This R34 proposes to develop a psychosocial intervention to help prevent and treat the increase in AUD risk and drinking following gastric bypass surgery. They will first work with patients to develop a computerized intervention that uses 2 (15 minute) sessions to target potential maladaptive behaviors that may lead to increased drinking or resuming drinking. The development plan is a good one following guidelines for stage I development which has two phases. Uses personalized text messaging to boost therapeutic effects following the initial treatment.

1. Significance

Strengths

- The increase in risk of AUD following bariatric surgery is a serious concern, the applicants make a good case, and their argument is in line with this reviewer's clinical experience. This issue is greatly important and needs to be addressed.
- A focused and personalized psychosocial intervention for addressing this seems to be a great idea in general and would make a great addition to the treatment options.
- The proposed type of brief intervention seems to have general face validity and might very well be cost effective.
- The computerized approach is a low burden one that may be easy to implement.

Weaknesses

- The theory (newly added mediational framework) behind the design of the intervention and expectations of its success seem somewhat loosely founded but at the same time they are certainly plausible.
- The claim that psychosocial interventions are the key to treating the condition following surgery is not really backed up. Why wouldn't medications be optimal for treating and preventing the increase in drinking?
- With the shift away from the more drastic bariatric surgeries (like roux-en-Y) to the more minimal versions like gastric band, might this decrease the problems with increased AUD risk somewhat? Since the banding procedure seems to have lower alcoholism risk…

2. Investigator(s)

Strengths

- Have the expertise needed for the work.
- The researchers are high quality and the addition of another expert brief intervention developer certainly will help.

Weaknesses

- None noted.

3. Innovation

Strengths

- I think the biggest strength here is in the targeting and adaptation to a specific population in need. It’s precision medicine in that regard.

Weaknesses

- Though it uses a valid and well-founded treatment approach, it’s not particularly innovative in that regard. mHealth would seem to be more the standard for this type of intervention these days than it is cutting edge.

4. Approach

Strengths

- In general, the overall development plan seems quite reasonable for this R34 project.
- The 2 currently proposed computerized sessions seem quite appropriate. I like the inclusion of content focused on addiction transfer which seems quite relevant.
- Follows a standard stage 1 development plan; phase 1 includes interview of 20 patients to develop and refine the intervention, followed by a 10-person test run and further adjustments. Then phase 2 is an N=60-person test run with comparison to TAU.

Weaknesses

- It’s unclear if the focus on change is appropriate for the population versus other possible foci like maintaining change.
- Will the type of surgery received (e.g., roux-en-Y, band, other) be accounted for in the analysis at all?
- I would think there are other defining features of this population that could lend themselves to assisting the targeted precision intervention approach, thus strengthening the approach.

5. Environment

Strengths

- Given the large number of bypass surgeries done in the Henry Ford system, this seems to be an ideal setting. Recruitment should be feasible.

Weaknesses

- None noted.

Study Timeline

Strengths

- Appropriate.

Weaknesses

- None noted.

Protections for Human Subjects

Acceptable Risks and/or Adequate Protections
Data and Safety Monitoring Plan (Applicable for Clinical Trials Only):
Acceptable

Inclusion Plans

- Sex/Gender: Distribution justified scientifically.
- Race/Ethnicity: Distribution justified scientifically.
- Inclusion/Exclusion Based on Age: Distribution justified scientifically.

Resource Sharing Plans:

Acceptable

Authentication of Key Biological and/or Chemical Resources:

Not Applicable (No Relevant Resources)

Budget and Period of Support

Recommend as Requested

CRITIQUE 3

Significance: 3
Investigator(s): 2
Innovation: 4
Approach: 3
Environment: 2

Overall Impact: This is an amended R34 application which was not discussed in the previous submission. The goal of the application is to optimize a technology-based intervention (computerized brief intervention; CBI and follow-up tailored text messaging) for patients who undergo bariatric surgery and to examine feasibility and acceptability of the intervention. Using standard behavioral treatment development procedures (i.e., stage model), the first phase focuses on identifying patient preferences and acceptability. The second phase consists of a randomized trial comparing the intervention to treatment as usual control group. The study will follow patients for 3 months post treatment. The original critique raised issues about the intervention being only minimally innovative, the justification for the iterative intervention model to be lacking, and the focus on intervention content to be insufficient. The question of why not using a standard intervention was also raised. The team responded to several of these concerns and has generally improved the application, yet the issue of impact remains salient and the application is overall deemed of modest impact. Specifically, there was a greater focus on content (education/norms and coping) and benchmarks by SAMHSA have been added. A mediational framework now suggests the intervention effects will be reached through changes in attitudes about alcohol use. Clarification about follow-up times, inclusion/exclusion criteria, and treatment goal (i.e., non-abstinence), have also improved technical aspects of the study. Overall, the primary question of what is unique and impactful to this intervention as compared to established/available interventions (e.g., Rethinking Drinking/Take Control) remains salient in providing an overall appraisal of this application. In other words, the successful completion of this application is unlikely to lead to a

significant step forward in the treatment of AUD as the bariatric surgery patients may benefit from standard counseling and from interventions that are already available.

Study Timeline

Strengths

- Appropriate.

Weaknesses

- None.

Protections for Human Subjects

Acceptable Risks and/or Adequate Protections

Data and Safety Monitoring Plan (Applicable for Clinical Trials Only):

Acceptable

Inclusion Plans

- Sex/Gender: Distribution justified scientifically.
- Race/Ethnicity: Distribution justified scientifically.
- Inclusion/Exclusion Based on Age: Distribution justified scientifically.

Resource Sharing Plans:

Acceptable

Authentication of Key Biological and/or Chemical Resources:

Not Applicable (No Relevant Resources)

Budget and Period of Support

Recommend as Requested

THE FOLLOWING SECTIONS WERE PREPARED BY THE SCIENTIFIC REVIEW OFFICER TO SUMMARIZE THE OUTCOME OF DISCUSSIONS OF THE REVIEW COMMITTEE, OR REVIEWERS' WRITTEN CRITIQUES, ON THE FOLLOWING ISSUES:

PROTECTION OF HUMAN SUBJECTS: UNACCEPTABLE

Some reviewers noted that the application does not provide a procedure to report unexpected or adverse events.

INCLUSION OF WOMEN PLAN: ACCEPTABLE

INCLUSION OF MINORITIES PLAN: ACCEPTABLE

INCLUSION ACROSS THE LIFESPAN: ACCEPTABLE

COMMITTEE BUDGET RECOMMENDATIONS: The budget was recommended as requested.

Footnotes for 1 R34 AA027775-01A1; PI Name: Matero, Lisa

NIH has modified its policy regarding the receipt of resubmissions (amended applications).See Guide Notice NOT-OD-18-197 at https://grants.nih.gov/grants/guide/notice-files/NOT-OD-18-197.html. The impact/priority score is calculated after discussion of an application by averaging the overall scores (1-9) given by all voting reviewers on the committee and multiplying by 10. The criterion scores are submitted prior to the meeting by the individual reviewers assigned to an application, and are not discussed specifically at the review meeting or calculated into the overall impact score. Some applications also receive a percentile ranking. For details on the review process, see http://grants.nih.gov/grants/peer_review_process.htm#scoring.

MEETING ROSTER

Clinical, Treatment and Health Services Research Review Subcommittee
National Institute on Alcohol Abuse and Alcoholism Initial Review Group
NATIONAL INSTITUTE ON ALCOHOL ABUSE AND ALCOHOLISM
AA-3
03/12/2020

Notice of NIH Policy to All Applicants: Meeting rosters are provided for information purposes only. Applicant investigators and institutional officials must not communicate directly with study section members about an application before or after the review. Failure to observe this policy will create a serious breach of integrity in the peer review process, and may lead to actions outlined in NOT-OD-14-073 at https://grants.nih.gov/grants/guide/notice-files/NOT-OD-14-073.html and NOT-OD-15-106 at https://grants.nih.gov/grants/guide/notice-files/NOT-OD-15-106.html, including removal of the application from immediate review.

CHAIRPERSON(S)

MIRANDA, ROBERT JR., PHD
PROFESSOR
DEPARTMENT OF PSYCHIATRY & HUMAN BEHAVIOR
CENTER FOR ALCOHOL & ADDICTION STUDIES
BROWN UNIVERSITY
PROVIDENCE, RI 02906

MEMBERS

ARIAS, ALBERT JOSEPH, MD
ASSOCIATE PROFESSOR
DEPARTMENT OF PSYCHIATRY
DIVISION OF ADDICTION PSYCHIATRY
VIRGINIA COMMONWEALTH UNIVERSITY
RICHMOND, VA 23284

BATES, MARSHA E., PHD
DISTINGUISHED PROFESSOR
DEPARTMENT OF KINESIOLOGY AND HEALTH
DIRECTOR, CARDIAC NEUROSCIENCE LABORATORY
CENTER OF ALCOHOL STUDIES
RUTGERS STATE UNIVERSITY OF NEW JERSEY
PISCATAWAY, NJ 08854-8001

BRADLEY, KATHARINE ANTHONY, MD
SENIOR INVESTIGATOR
WASHINGTON HEALTH RESEARCH INSTITUTE
KAISER PERMANENTE
SEATTLE, WA 98101

CARROLL, KATHLEEN M., PHD
ALBERT E. KENT PROFESSOR OF PSYCHIATRY
DIRECTOR OF PSYCHOSOCIAL RESEARCH
DIVISION OF ADDICTIONS
DEPARTMENT OF PSYCHIATRY
YALE UNIVERSITY SCHOOL OF MEDICINE
WEST HAVEN, CT 06511

CHILDS, EMMA, PHD
ASSOCIATE PROFESSOR
DEPARTMENT OF PSYCHIATRY
THE UNIVERSITY OF ILLINOIS AT CHICAGO
CHICAGO, IL 60637

CHOI, DOO-SUP, PHD
PROFESSOR OF PHARMACOLOGY AND PSYCHIATRY
DEPARTMENT OF MOLECULAR PHARMACOLOGY
AND EXPERIMENTAL THERAPEUTICS
MAYO CLINIC COLLEGE OF MEDICINE
ROCHESTER, MN 55905

CURRY, JOHN F, PHD
PROFESSOR
DEPARTMENT OF PSYCHOLOGY AND NEUROSCIENCE
DUKE UNIVERSITY
DURHAM, NC 27705-5696

DEVINE, ERIC G., PHD
ASSISTANT PROFESSOR OF PSYCHIATRY & DIRECTOR
CLINICAL STUDIES UNIT
DEPARTMENT OF PSYCHIATRY
BOSTON UNIVERSITY SCHOOL OF MEDICINE
BOSTON, MA 02118

KELLY, JOHN F., PHD
ELIZABETH R. SPALLIN PROFESSOR OF PSYCHIATRY
DIRECTOR, RECOVERY RESEARCH INSTITUTE
ASSOCIATE DIRECTOR, CENTER FOR ADDICTION MEDICINE
MASSACHUSETTS GENERAL HOSPITAL
BOSTON, MA 02114

KING, KEVIN MICHAEL, PHD
ASSOCIATE PROFESSOR
DEPARTMENT OF PSYCHOLOGY
UNIVERSITY OF WASHINGTON
SEATTLE, WA 98195

RAY, LARA A., PHD
PROFESSOR
DEPARTMENT OF PSYCHOLOGY
UNIVERSITY OF CALIFORNIA, LOS ANGELES
LOS ANGELES, CA 90095

SCIENTIFIC REVIEW OFFICER

ESPINOZA, LUIS, PHD
SCIENTIFIC REVIEW OFFICER
EXTRAMURAL PROJECT REVIEW BRANCH
NATIONAL INSTITUTE ON ALCOHOL ABUSE AND ALCOHOLISM
NATIONAL INSTITUTES OF HEALTH
BETHESDA, MD 20817

SRINIVAS, RANGA V., PHD
CHIEF, EXTRAMURAL PROJECT REVIEW BRANCH
EXTRAMURAL PROJECT REVIEW BRANCH
NATIONAL INSTITUTE ON ALCOHOL ABUSE AND ALCOHOLISM
NATIONAL INSTITUTES OF HEALTH
BETHESDA, MD 20892

EXTRAMURAL SUPPORT ASSISTANT

STRINGFIELD, DONNA
EXTRAMURAL SUPPORT ASSISTANT
OFFICE OF EXTRAMURAL ACTIVITIES
NATIONAL INSTITUTE ON ALCOHOL ABUSE AND ALCOHOLISM
NATIONAL INSTITUTES OF HEALTH
ROCKVILLE, MD 20852

Consultants are required to absent themselves from the room during the review of any application if their presence would constitute or appear to constitute a conflict of interest.
